# Pathological complete response as a surrogate for relapse-free survival in patients with triple negative breast cancer after neoadjuvant chemotherapy

**DOI:** 10.18632/oncotarget.9369

**Published:** 2016-05-14

**Authors:** JunJie Li, Sheng Chen, CanMing Chen, GenHong Di, GuangYu Liu, Jiong Wu, ZhiMin Shao

**Affiliations:** ^1^ Department of Breast Surgery, Fudan University Shanghai Cancer Center, Shanghai, Peoples Republic of China; ^2^ Department of Oncology, Shanghai Medical College, Fudan University, Shanghai, Peoples Republic of China

**Keywords:** breast cancer, neoadjuvant, pathological complete response, triple negative

## Abstract

We retrospective analyzed triple negative breast cancer (TNBC) patients who received either taxane-based or anthracycline-based neoadjuvant chemotherapy, evaluated whether pathological complete response (pCR) is a surrogate endpoint for relapse free survival (RFS) in TNBC and explored which subgroup of patients benefits more from superior treatment regimen. 186 patients received taxane-based (Group A) or anthracycline-based (Group B) neoadjuvant chemotherapy, median follow-up was 48.1 months. 42 patients received total pCR (ypT0/is ypN0), 34 in Group A and 8 in Group B, *p* < 0.001. Patients who achieved pCR had an increased RFS when compared with non-pCR patients, *p* = 0.043. Patients in Group A had a better RFS, *p* = 0.025, after adjusting for tumor size and clinical lymph node status before neoadjuvant therapy. Only patients sensitive to neoadjuvant chemotherapy exhibited RFS benefit from taxane-based treatment, and those who were treatment insensitive had similar RFS between both groups. Our analysis showed Taxane-based regimen had higher pCR rate and could predict improved RFS in TNBC, and the prognostic value was greater in treatment sensitive patients. This retrospective analysis supports the use of pCR as a surrogate endpoint for RFS in TNBC.

## INTRODUCTION

Neoadjuvant systemic therapy is frequently adopted to reduce the size and extent of locally advanced tumors, and it aims to render locally advanced cancers operable and facilitate breast-conserving surgery.[1] Currently, it is widely used for the *in vivo* assessment of drug efficacy and could expedite the development and approval of treatments for early breast cancer.[2] Anthracycline followed by taxane showed a survival benefit over anthracycline alone in the adjuvant setting.[3, 4] When these regimens were compared in the neoadjuvant setting, such as the NSABP B27 trial, an increase of the pathological complete response (pCR) rate of the sequential regimen was found.[5] Afterwards, a large number of neoadjuvant trials were conducted to obtain a quantifiable evaluation of the sensitivity or resistance of treated patients, and pCR has been the most commonly used surrogate endpoint to predict the survival benefit, as pCR is associated with better survival than residual diseases.[6] Then we formed the hypothesis that regimens with an increase in the frequency of pCR may lead to better outcomes.

Recent data from the neoALLTO trial showed that the pCR rate was significantly increased when adding lapatinib to trastuzumab-based neoadjuvant treatments in HER2-positive tumors.[7] However, event-free survival and overall survival did not differ between treatment groups after 3.77 years of follow-up, and the combination regimen also showed no survival benefit in the ALLTO trial.[8] The hypothesis that an increase in response with the addition of a new agent to standard therapy in the neoadjuvant setting will predict a survival benefit in the early breast cancer setting was proposed. Soon thereafter, a US Food and Drug Administration (FDA) meta-analysis failed to show significant improvement in event-free survival or overall survival related to improved pCR rates in most included trials.[9] Additionally, a meta-regression of 29 studies did not support the use of pCR as a surrogate end point for survival.[10] Therefore, the FDA recommended that accelerated approval can be based on an improved pCR rate, but improved event-free survival remains the end point for full approval.[11]

Triple negative breast cancer (TNBC) accounts for 15% of all breast cancers. It is an aggressive subtype defined by the absence of the expression and/or amplification of estrogen and progesterone receptor (ER/PR) as well as HER2, preventing the use of currently available endocrine therapy and/or HER2-directed drugs.[12]

TNBC has specific molecular features that could be possible targets for new biologically targeted drugs.[13] However, to date, no single targeted therapy has been approved, and cytotoxic chemotherapy currently remains the main therapy. As we know, TN patients are more likely to obtain pCR compared with non-TN patients, and TN patients who achieved pCR showed a statistically significant improvement in clinical benefit.[9]

Therefore, we tend to administer neoadjuvant chemotherapy to TNBC patients. It is quite important to identify whether pCR can be a surrogate endpoint in neoadjuvant trials for TNBC, to define a better regimen and to guide future trials. Here, we performed a retrospective analysis to assess whether a regimen with an increased pCR rate can lead to a better survival in patients with TNBC and to explore which subgroup of patients benefits more from the superior regimen.

## MATERIALS AND METHODS

Patients with TNBC were retrospectively analyzed to assess whether pCR ais a surrogate endpoint of relapse-free survival (RFS). All cases were treated in Shanghai Cancer Center, Fudan University, and the majority of the cases were extracted from three prospective, single-arm, unicentric phase II trials conducted in Shanghai Cancer Center. The results of these three trials have been published elsewhere.[14–16] As a secondary aim, we explored which subgroup of patients benefits more from the superior regimen with a higher pCR rate.

### Patients’ eligibility criteria

**Figure 1:**
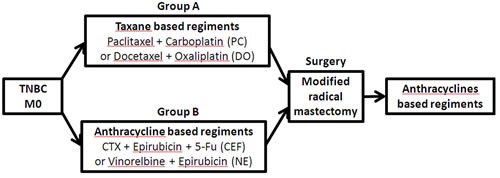
Study design Taxane-based chemotherapy includes PC (paclitaxel and carboplatin) and DO (docetaxel and oxaliplatin). Anthracycline-based neoadjuvant chemotherapy includes CEF (cyclophosphamide, epirubicin, 5- fluorouracil) and NE (vinorelbine and epirubicin).

All the patients received neoadjuvant chemotherapy in our institution from January 2000 to December 2012, with core needle biopsy (CNB)-diagnosed invasive breast cancer and a immunohistochemical (IHC) report of the CNB tissue-confirmed TN phenotype, defined as ER < 1% positive, PR < 1% positive, HER2 0 or 1-2+ with FISH negativity. Patients received 3-4 cycles of anthracycline-based neoadjuvant chemotherapy, such as CEF (cyclophosphamide, epirubicin, 5- fluorouracil) or NE (vinorelbine and epirubicin), or taxane-based chemotherapy, such as PC (paclitaxel and carboplatin) or DO (docetaxel and oxaliplatin). Patients who received both anthracycline- and taxane-based neoadjuvant regimens were excluded. Patients with metastatic disease before neoadjuvant chemotherapy were excluded. After neoadjuvant therapy, patients underwent surgical treatment in our center and detailed pathology reports were provided after surgery. Patients with breast-conserving therapy were excluded. All patients received mastectomy and axillary dissection. Patients received only anthracycline-based adjuvant chemotherapy (no taxanes). Patients who were ER and/or PR positive in post-operation pathology reports were administered endocrine therapy. Radiation therapy was conducted according to the features of the primary tumor (Figure 1).

### Data extraction and outcome measures

We extracted the following data from each patient: age at diagnosis (year), menstrual status, tumor size before neoadjuvant therapy, axillary lymph node (LN) status before neoadjuvant therapy, neoadjuvant chemotherapy regimens (type of chemotherapy, number of cycles), surgical pathology reports (including pathological tumor size, tumor grade, involvement of LN, ER and PR status, and HER2/neu status), additional postsurgical treatments (adjuvant chemotherapy, endocrine therapy and radiation), and follow-up information. The stage was determined from pathological records and classified according to the AJCC TNM guidelines. Axillary nodes negative before neoadjuvant chemotherapy were defined upon clinical physical examination and ultrasound-negative or clinically and/or ultrasound positive with a negative fine needle aspiration. IHC staining of ER, PR and Her-2/neu were carried out in the pathology department of our hospital. The work was performed according to established procedures described elsewhere. The definition of pCR is non-invasive cancer from the breast and nodes, ypT0/is ypN0.[11] The tumor shrinkage rate was defined as 1 minus the ratio of tumor size on the surgical pathology report to tumor size before neoadjuvant chemotherapy on the ultrasound report. RFS was defined as the time to the first relapse, not including second primary breast cancer or other malignant neoplasms, and was calculated from the date of surgery to the first evidence of recurrence (any site). Follow-up information was obtained from hospital and office records and from the patients and their families. The date of the last follow-up and the date of recurrence or death were recorded.

### Statistical methods

We categorized patients with taxane-based neoadjuvant therapy as Group A and patients with anthracycline-based neoadjuvant therapy as Group B. Age at diagnosis and tumor sizes were recorded as continuous variables, and the other variables were treated as categorical data. The significance of differences in categorical or continuous variables was evaluated by the chi-squared test and t-test, respectively. The actual probability of survival was estimated by the Kaplan-Meier product-limit method. The log-rank test was used to compare the survival curves. Multivariate analysis was carried out to assess the major significant prognostic factors on survival using the Cox proportional hazards regression model. Hazard ratios (HR) were presented with their 95% confidence intervals. A P-value less than or equal to 0.05 was considered statistically significant. All statistical analyses were performed using the SPSS statistical software package (version 16.0; SPSS Company, Chicago, IL).

## RESULTS

**Figure 2:**
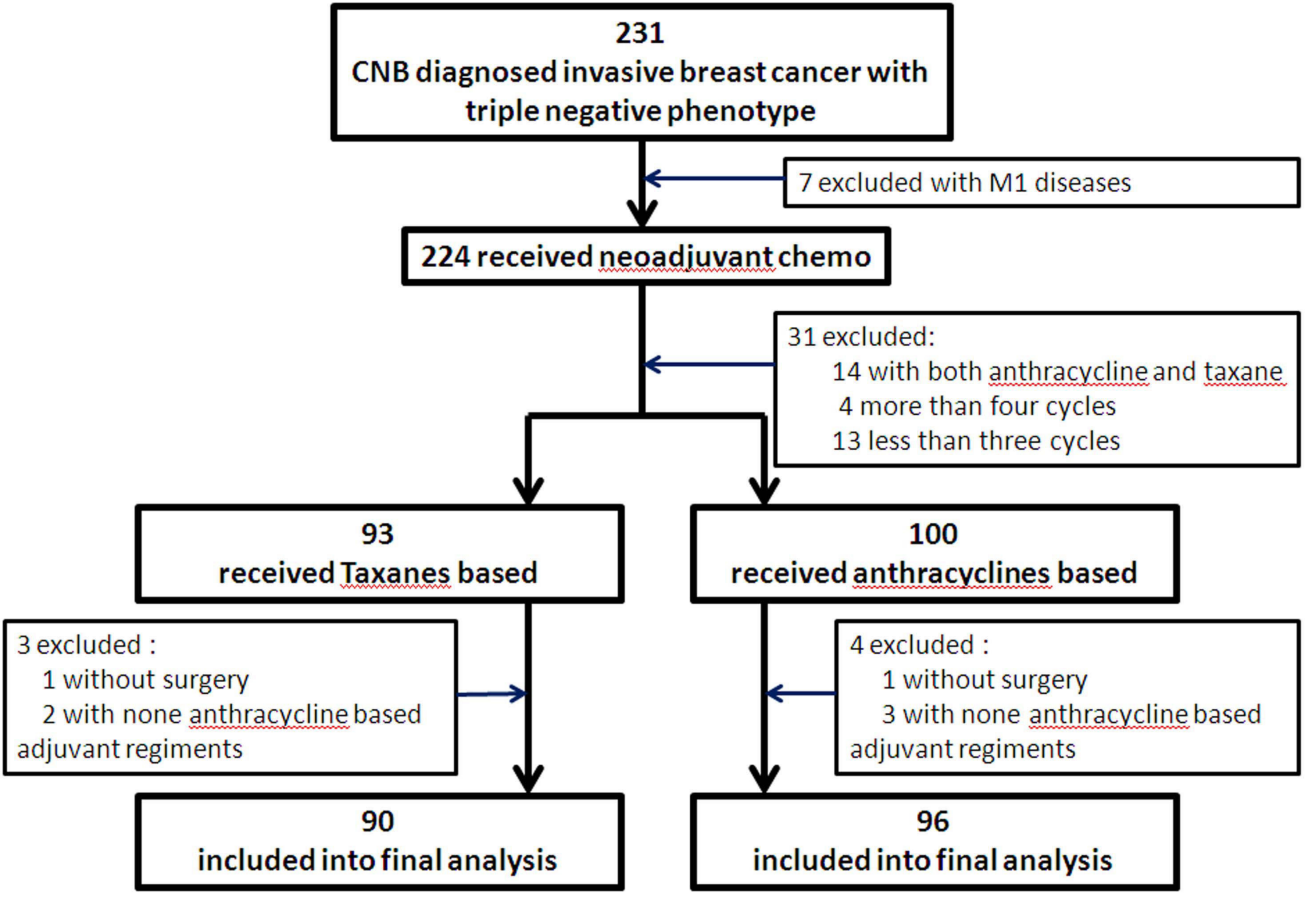
Patient selection and the analyzed profiles of patient subsets

We identified 231 patients with CNB-diagnosed invasive TNBC from January 2000 to December 2012. Among them, 7 had primary metastatic breast cancer and 31 received neoadjuvant chemotherapy but did not meet the eligibility criteria (14 with anthracycline combined with taxane, 4 with more than 4 cycles, and 13 with less than 3 cycles). 193 patients received either anthracycline- or taxane-based regimens, 2 did not undergo surgical operations, and 5 received no anthracycline-based regimens in the adjuvant setting. Therefore, there were 90 patients with taxane-based regimens (Group A) and 96 with anthracycline-based regimens (Group B) in the neoadjuvant setting included into the final analysis (Figure 2).

### Basic characteristics

The median follow-up time was 48.1 months. As shown in Table 1, the median age was similar between the two groups, 47.5 years old in Group A and 46.7 years old in Group B. Fifty-five patients (61%) in Group A and 68 (70.8%) in Group B were premenopausal at diagnosis. The median tumor size before neoadjuvant therapy was 5.6 cm in Group A and 6.6 cm in Group B, respectively. Patients in Group B had more T3-4 diseases (67% *vs*. 41%, *p* < 0.01) and more clinically positive lymph nodes (86.5% *vs*. 73.3%, *p* = 0.028) before neoadjuvant therapy. In Group A, 79 patients received PC and 11 received DO treatment regimens. In Group B, 47 patients received NE and 49 received CEF. 23 non-pCR patients had HR positive residual disease after neoadjuvant therapy, 4 in Group A and 19 in Group B. None of the patients turned out to be HER2-positive after neoadjuvant therapy. Approximately 74 patients in Group A and 86 patients in Group B received radiation after surgery.

**Table 1:** Patients’ basic clinical characteristics

	Group A	Group B	*p* Value
N	90	96	
Median Age	47.5(28-78)	46.7(24-73)	
Pre-Meno	55(61)	68(70.8)	0.167
Before Neoadjuvant			
T Size (cm)	5.6	6.6	0.136
T Stage			0.001
T1	4	0	
T2	45	29	
T3	36	63	
T4	5	4	
N-,%	24(26.7)	13(13.5)	
N+,%	66(73.3)	83(86.5)	
Regimens*	79 PC	47 NE	
	11 DO	49 CEF	
Post-operation			
HR+ %	4(4.4)	19(19.8)	0.002
Radiation %	74(82.2)	86(89.6)	0.204

* PC: paclitaxel and carboplatin; DO: docetaxel and oxaliplatin; CEF: cyclophosphamide, epirubicin, 5- fluorouracil; NE: vinorelbine and epirubicin.

### Response rate

Table 2 shows that the rate of non-invasive residual cancer in the breast (ypT0/is) was significantly higher in Group A, 44.4% *vs*. 9.4%, *p* < 0.001. The median T size after neoadjuvant chemotherapy was 1.24 cm in Group A, smaller than that in Group B, 2.98 cm, *p* = 0.052. Patients in Group A had fewer lymph nodes involved after neoadjuvant therapy than patients in Group B, 41.1% *vs*. 70.8%, *p* < 0.001. 42 patients received total pCR (ypT0/is ypN0), 34 (37.8%) in Group A and 8 (8.3%) in Group B, *p* < 0.001.

**Table 2:** Pathology response after neoadjuvant chemotherapy

	Group A	Group B	*p* Value
Post-surgery			
Median-T Size (cm)	1.24(0.9-1.58)	2.98(2.54-3.41)	0.052
pT Stage			
pT0 %	40 (44.4)	9 (9.4)	<0.001
pT1 %	30 (33.3)	34 (35.4)	
pT2 %	16 (17.8)	40 (41.7)	
pT3 %	4 (4.4)	13 (13.5)	
pN+,%	37 (41.1)	68 (70.8)	<0.001
Total pCR*	34(37.8)	8(8.3)	<0.001
Breast pCR**	40(44.4)	9(9.4)	<0.001

* Total pCR: non-invasive cancer from the breast and nodes, ypT0/is ypN0.

** Breast pCR: non-invasive cancer in breast, ypT0/is.

### Survival analysis

**Figure 3:**
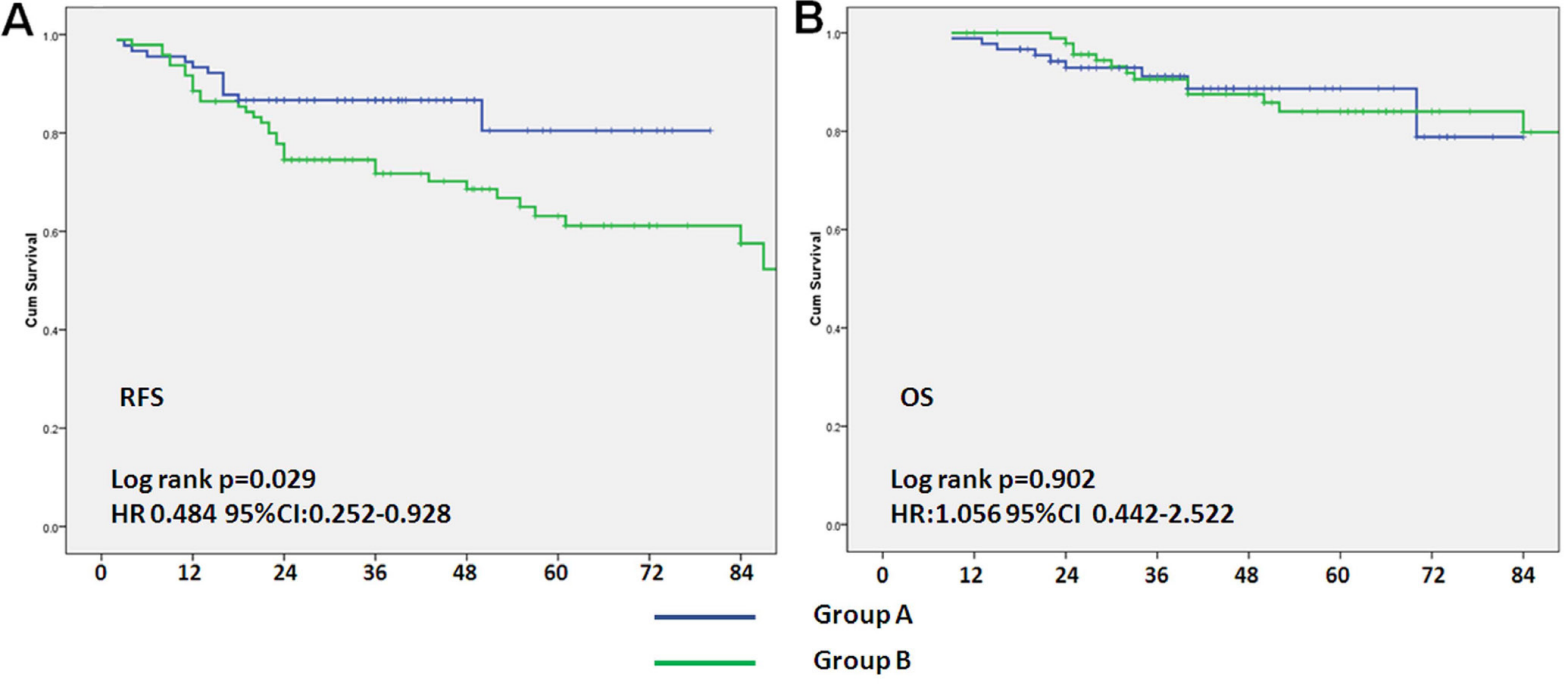
Association between neoadjuvant chemotherapy regimens and relapse-free survival and overall survival The blue solid line represents patients with taxane-based regimens (**A**); the green solid line represents patients with anthracycline-based regimens (**B**). Adjusted for tumor size (T1-2 *vs*. T3-4) and clinical lymph node status (positive *vs*. negative) before neoadjuvant. HR: hazard ratio.

**Figure 4:**
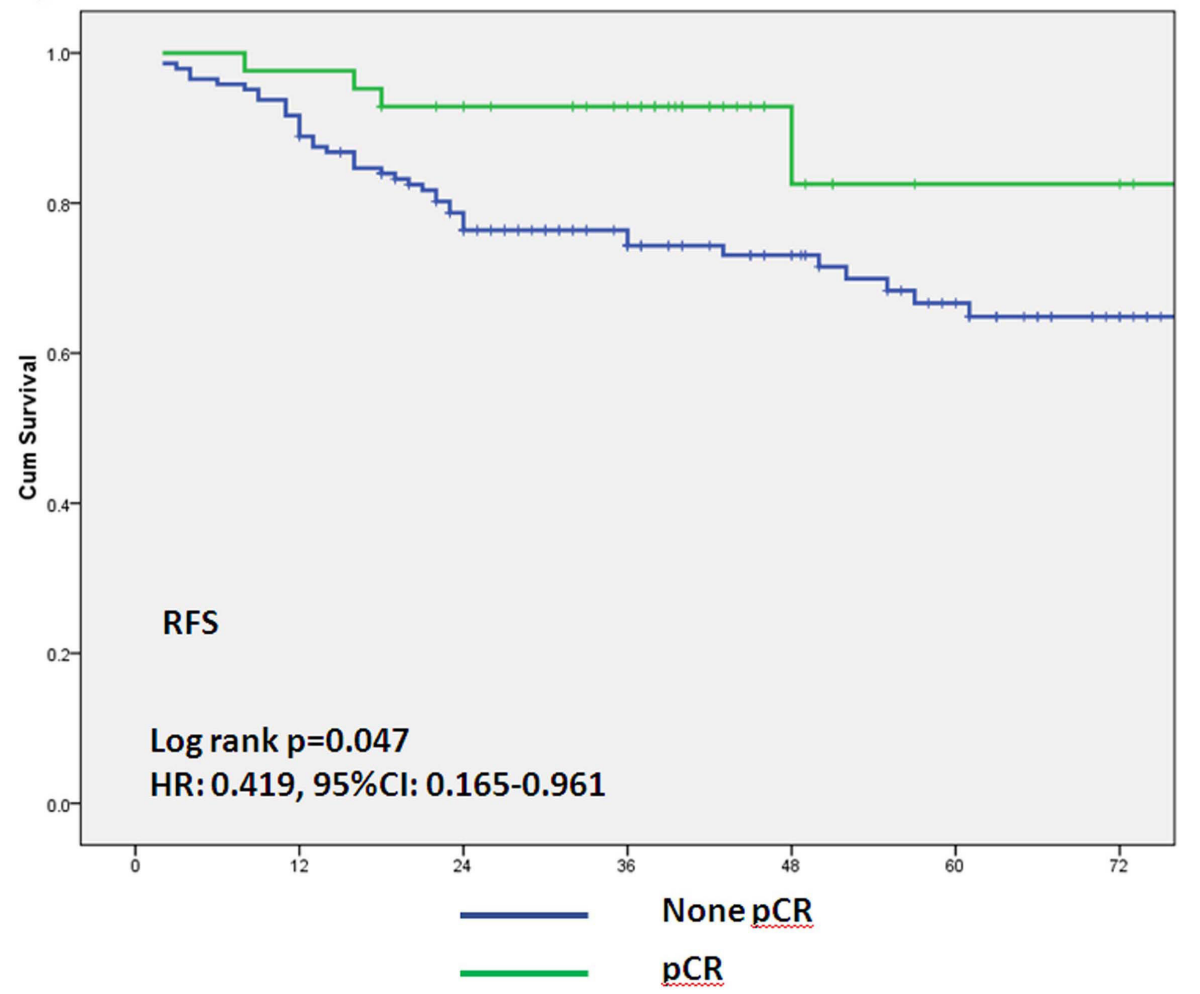
Association between pathological complete response and relapse-free survival The green solid line represents patients with pathological complete response (ypT0/is ypN0); the blue solid line represents patients with no pathological complete response.

After median follow-up 4 years, 13 relapsed and 9 died in Group A and 36 relapsed and 14 died in Group B. Kaplan- Meier survival curves (Figure 3) showed that patients in Group A had a better RFS, *p* = 0.029 (HR: 0.484, 95%CI 0.252-0.928), after adjustment for tumor size (T1-2 *vs*. T3-4) and clinical lymph node status (positive *vs*. negative) before neoadjuvant therapy. No difference was found in overall survival, *p* = 0.902. Overall, patients achieved pCR had increased RFS when compared with non-pCR patients, *p* = 0.043, HR: 0.419, 95%CI: 0.165-0.961, (Figure 4). Among pCR patients, only 2 in Group A and 3 in Group B had disease recurrence in the follow-up period. We defined tumors sensitive to neoadjuvant chemotherapy according to tumor shrinkage rates, and divided into different levels, with shrinkage rates ≥75%, ≥50%, ≥25% or < 25%. As shown in Table 3, more patients in Group A were regarded as treatment sensitive, and a trend toward RFS benefit with taxane-based regimens across all the levels in tumor “sensitive” patients, with HRs of 0.37, 0.44, and 0.414, respectively. The RFS of those insensitive tumors were similar, with HRs from 0.883 to 1.134 and no statistically significant differences between the two groups, *p* > 0.05.

**Table 3:** RFS according to tumor shrinkage rate

Tumor Shrink Rate	Taxane 4y RFS (n)	Anthracycline 4y RFS (*n*)	*p* value	HR	95%CI
Sensitive					
≥75%	92%(63)	79%(28)	0.046	0.37	0.126-0.886
≥50%	87%(74)	78%(60)	0.067	0.44	0.177-1.011
≥25%	85%(83)	76%(75)	0.036	0.414	0.182-0.944
Insensitive					
<75%	67%(27)	66%(68)	0.854	0.924	0.396-2.152
<50%	63%(16)	61%(36)	0.789	0.883	0.355-2.195
<25%	61%(7)	57%(21)	0.816	1.134	0.393-3.270

Tumor shrinkage rate: defined as 1 minus the ratio of tumor size on the surgical pathology report to the tumor size before neoadjuvant chemotherapy on the ultrasound report.

## DISCUSSION

In the current retrospective analysis, we found taxane-based neoadjuvant chemotherapy regimen produced an increase in the frequency of pCR and could predict improved RFS in TNBC.

We defined TNBC according to CNB tissues. Previous studies reported that a discordance of ER, PR and HER2 status between CNB and surgical excision tissues existed, suggesting that results from CNB should be used with caution. [17] Contrarily, a meta-analysis indicated that CNB has high diagnostic accuracy in evaluating ER, PR, and HER2 status.[18] In fact, current neoadjuvant treatment strategies are mainly based on the presence of ER, PR and HER2 status through CNB prior to treatment. Receptor status may also differ between CNB and the residual tumor after surgery for breast cancer treated with neoadjuvant chemotherapy. ER and PR discordance was detected in 10-30% of the patients in the neoadjuvant chemotherapy group, while HER2 amplification tested by FISH had good concordance.[19, 20] In our study, we found 23 non-pCR patients had HR positive diseases in post-operation pathology reports, and recommended these patients undergo adjuvant endocrine therapy. Fewer patients in the taxane-based group had HR positive diseases after surgery, possibly due to higher pCR rates in the taxane-based group, hence, if we just calculated the HR change rate in non-pCR patients, it was 4 in 50 patients in group A and 19 in 87 patients in group B. On the other hand, the residue HR positive diseases may be less sensitive to anthracycline based treatment, led to a worse outcome in group B.

We selected patients who either received anthracycline-based or taxane-based neoadjuvant treatment as the study groups. All treatments were conducted for 3-4 cycles before surgery. Regarding adjuvant therapy, to minimize all other treatment variability, we included patients with the same surgical approach (mastectomy and axillary dissection), the same postoperative systemic therapy (only anthracycline-based regimens) and the same criteria for the delivery of radiotherapy. Therefore, the difference of RFS observed in our study was mainly due to the different neoadjuvant chemotherapy treatments. However, the prescription of anthracycline to patients who had limited benefit from anthracycline neoadjuvant therapy may correlate with the worse outcome in Group B.

The definition of pCR used in our study was non-invasive cancer from the breast and nodes (ypT0/is ypN0), which is regarded as the preferred definition that correlates best with long-term outcome. [9, 21] We found anthracycline-based group had a pCR of 8.3%, similar with the results of NSABP B18 and NSABP B27 trials that 4 cycles of neoadjuvant AC had a pCR rate of 9% and 13%, although the definition of pCR in these trials was non-invasive cancer in breast, and the results were not limited to TNBC.[5, 22] The pCR rate was 37.8% in the taxane-based group, consistent with our previous studies that the pCR rate was 34.48% for the DO regimen and 33.3% for the PC regimen in TNBC.[15, 16]. The relatively low pCR rate in the anthracycline-based group may due to either NE or CEF used was dose-dense, and the dose for epirubicin was 75 mg/m^2^ every 3 weeks. And higher pCR rate in group A may also due to the combination used of platinum with taxane, as recent studies GeparSixto and CALGB40603 indicated that adding platinum may significantly increase pCR rate in TNBC.[23, 24]

Whether pCR is a surrogate endpoint of survival in TNBC is still unknown, according to the controversial results of GeparSixto and CALGB40603.[23, 24] The challenge is that RFS benefits are highly dependent upon the magnitude of pCR benefit and prognosis in pCR *vs*. non-pCR cases. A large difference in pCR is required to see even a small difference in RFS, assuming that a 20% increase in pCR leads to a 5% increase in survival. [25] In the current analysis, there was a huge difference in the pCR between Group A and Group B, 37.8% *vs*. 8.3%, *p* < 0.001. And patients with pCR had significantly increased in RFS, *p* = 0.043, HR = 0.419. Therefore, a positive result was recorded. We concluded that the increase in pCR with a taxane-based regimen translated to a survival benefit. Of note, our study is not a randomized study, the basic clinical-pathology characteristics were not well balanced. Patients in Group B had larger tumors and more nodes involved, which may result to the lower pCR rate and worse RFS. Hence, we used Cox proportional hazards regression model to compare the outcome between two groups and adjusted for tumor size and node status.

**Figure 5:**
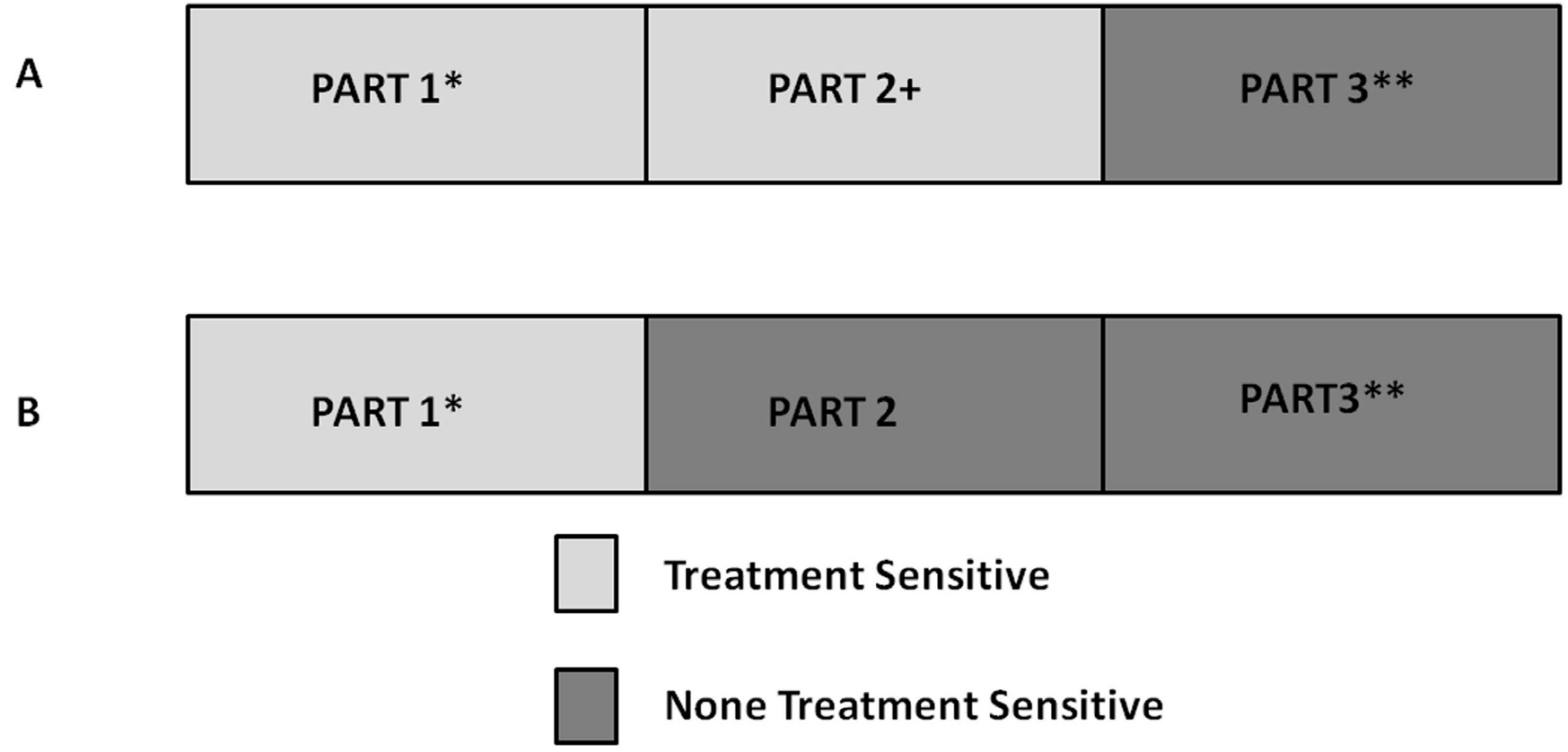
Screening value of neoadjuvant chemotherapy Part 1 represents patients who are sensitive to both treatments and have better outcomes. Part 3 represents patients who are not sensitive to either treatment and have worse outcomes. A more effective regimen moves those “Part 2” patients from the treatment-insensitive group to the treatment-sensitive group as “Part 2+.”

We evaluated what type of patients might benefit more from the taxane-based regimens. No previous studies have provided a clear definition of “chemotherapy-sensitive tumors.” Here, we defined a tumor to be sensitive to neoadjuvant chemotherapy based on the tumor shrinkage rate. Interestingly, patients sensitive to neoadjuvant chemotherapy (no matter the cut-off value of the tumor shrinkage rate) had a trend of RFS benefit from taxane-based treatment. The HRs in different levels were quite similar, from 0.37 to 0.44, and those that were treatment-insensitive had similar outcomes, regardless of the treatment regimens. Our results indicate that responsive tumors may have actually achieved increased survival through a superior neoadjuvant regimen, which means a superior regimen could screen out more sensitive patients and provide a survival benefit for this particular subgroup of patients. Neoadjuvant treatment serves as a screening method, as shown in Figure 5, “Part 1” represents patients who are sensitive to both treatments and have better outcomes, while “Part 3” represents patients who are insensitive to either treatments and have worse outcomes. A more effective regimen moves “Part 2” patients from the treatment-insensitive to the treatment-sensitive group as “Part 2+,” improving their survival. Whether a regimen is superior or not depends on the proportion of patients in “Part 2” and the survival difference between “sensitive” and “insensitive” patients. The goal of neoadjuvant clinical trials is to test new treatment strategies in previously treatment-insensitive patients and to evaluate the number of patients who the treatment moves to the “Part 2+” group.

Our results further increase the evidence that pCR as a surrogate for RFS in patients with TNBC after neoadjuvant chemotherapy. Further trials can use pCR as a key endpoint to assess drug efficacy and select a new treatment strategy. Further studies should also focus on those non-treatment sensitive tumors to search for regimens that target new mechanisms and/or combine more effective cytotoxic drugs, which may turn treatment-insensitive tumors into sensitive ones and improve these patients’ outcomes.

No Funding.

Ethical approval: All procedures performed in studies involving human participants were in accordance with the ethical standards of the institutional and/or national research committee and with the 1964 Helsinki declaration and its later amendments or comparable ethical standards.

This article does not contain any studies with animals performed by any of the authors.

Informed consent: Informed consent was obtained from all individual participants included in the study.
